# Identification of platelet-related subtypes and diagnostic markers in pediatric Crohn’s disease based on WGCNA and machine learning

**DOI:** 10.3389/fimmu.2024.1323418

**Published:** 2024-02-14

**Authors:** Dadong Tang, Yingtao Huang, Yuhui Che, Chengjun Yang, Baoping Pu, Shiru Liu, Hongyan Li

**Affiliations:** ^1^ Clinical Medical College, Chengdu University of Traditional Chinese Medicine, Chengdu, China; ^2^ First Clinical Medical College, Liaoning University of Traditional Chinese Medicine, Shenyang, China; ^3^ Department of Otorhinolaryngology, Zigong Hospital of Traditional Chinese Medicine, Zigong, China; ^4^ Anorectal Disease Department, Hospital of Chengdu University of Traditional Chinese Medicine, Chengdu, China

**Keywords:** pediatric Crohn’s disease, platelet, immune infiltration, machine learning, bioinformatics

## Abstract

**Background:**

The incidence of pediatric Crohn’s disease (PCD) is increasing worldwide every year. The challenges in early diagnosis and treatment of PCD persist due to its inherent heterogeneity. This study’s objective was to discover novel diagnostic markers and molecular subtypes aimed at enhancing the prognosis for patients suffering from PCD.

**Methods:**

Candidate genes were obtained from the GSE117993 dataset and the GSE93624 dataset by weighted gene co-expression network analysis (WGCNA) and differential analysis, followed by intersection with platelet-related genes. Based on this, diagnostic markers were screened by five machine learning algorithms. We constructed predictive models and molecular subtypes based on key markers. The models were evaluated using the GSE101794 dataset as the validation set, combined with receiver operating characteristic curves, decision curve analysis, clinical impact curves, and calibration curves. In addition, we performed pathway enrichment analysis and immune infiltration analysis for different molecular subtypes to assess their differences.

**Results:**

Through WGCNA and differential analysis, we successfully identified 44 candidate genes. Following this, employing five machine learning algorithms, we ultimately narrowed it down to five pivotal markers: GNA15, PIK3R3, PLEK, SERPINE1, and STAT1. Using these five key markers as a foundation, we developed a nomogram exhibiting exceptional performance. Furthermore, we distinguished two platelet-related subtypes of PCD through consensus clustering analysis. Subsequent analyses involving pathway enrichment and immune infiltration unveiled notable disparities in gene expression patterns, enrichment pathways, and immune infiltration landscapes between these subtypes.

**Conclusion:**

In this study, we have successfully identified five promising diagnostic markers and developed a robust nomogram with high predictive efficacy. Furthermore, the recognition of distinct PCD subtypes enhances our comprehension of potential pathogenic mechanisms and paves the way for future prospects in early diagnosis and personalized treatment.

## Introduction

Crohn’s disease is a chronic inflammatory disorder primarily affecting the gastrointestinal tract, and it is categorized as an inflammatory bowel disease (IBD) along with ulcerative colitis. Crohn’s disease can involve any part of the gastrointestinal tract, from the oral cavity to the rectum, but most commonly affects the small intestine and the initial portion of the large intestine (1). In recent years, with the change in people’s lifestyles and the influence of environmental factors, the incidence and prevalence of Crohn’s disease have gradually increased and shown a significant trend toward younger ages (2). Pediatric Crohn’s disease (PCD) patients usually have more extensive involvement, a more severe disease, and more atypical symptoms than adults with Crohn’s disease (3). However, PCD is not the early stage of adult Crohn’s disease and is quite different from adult IBD etiology and symptoms (4, 5). Presently, the precise etiology of PCD remains partially elusive. Generally, it is attributed to factors such as environmental influences, alterations in intestinal flora, genetic predisposition, anomalous immune responses of the mucosa, and impairment of epithelial barrier function (1, 6).

Given the heterogeneity of PCD patient characteristics, the diagnosis of PCD is particularly difficult in clinical practice and often requires a combination of ultrasound, endoscopy, and pathology to confirm the diagnosis (7). Therefore, it is imperative to find more accurate, convenient, non-invasive, and highly specific diagnostic tools for children with Crohn’s disease. In recent years, molecular typing has made great strides in the understanding and treatment of many diseases. Mature molecular typing strategies are able to predict the optimal therapeutic strategy prior to patient treatment, thereby substantially improving patient prognosis (8). In previous reports, the molecular subtyping of diseases such as hepatocellular carcinoma, osteoarthritis, and rheumatoid arthritis, for example, has laid the foundation for their individualized treatment (9–11). Therefore, the development of molecular subtyping of PCD based on its molecular features is crucial for improving the accuracy of clinical treatment decisions and deepening our understanding of PCD.

Platelets are small blood cells derived from bone marrow, and in addition to their well-known role in blood clotting and wound healing, there is growing evidence that they also play a crucial role in autoimmune processes (12, 13). Currently, research on the role of platelets in the pathogenesis of a number of autoimmune diseases, including rheumatoid arthritis and systemic lupus erythematosus, is progressing (14, 15). However, the contribution of platelets to the pathogenesis of PCD, a classic autoinflammatory disease, has not been investigated. Expanding the scope to encompass systems and cell types that have not been extensively studied could furnish a more comprehensive understanding of PCD progression and potentially uncover novel biomarkers.

**Figure 1:**
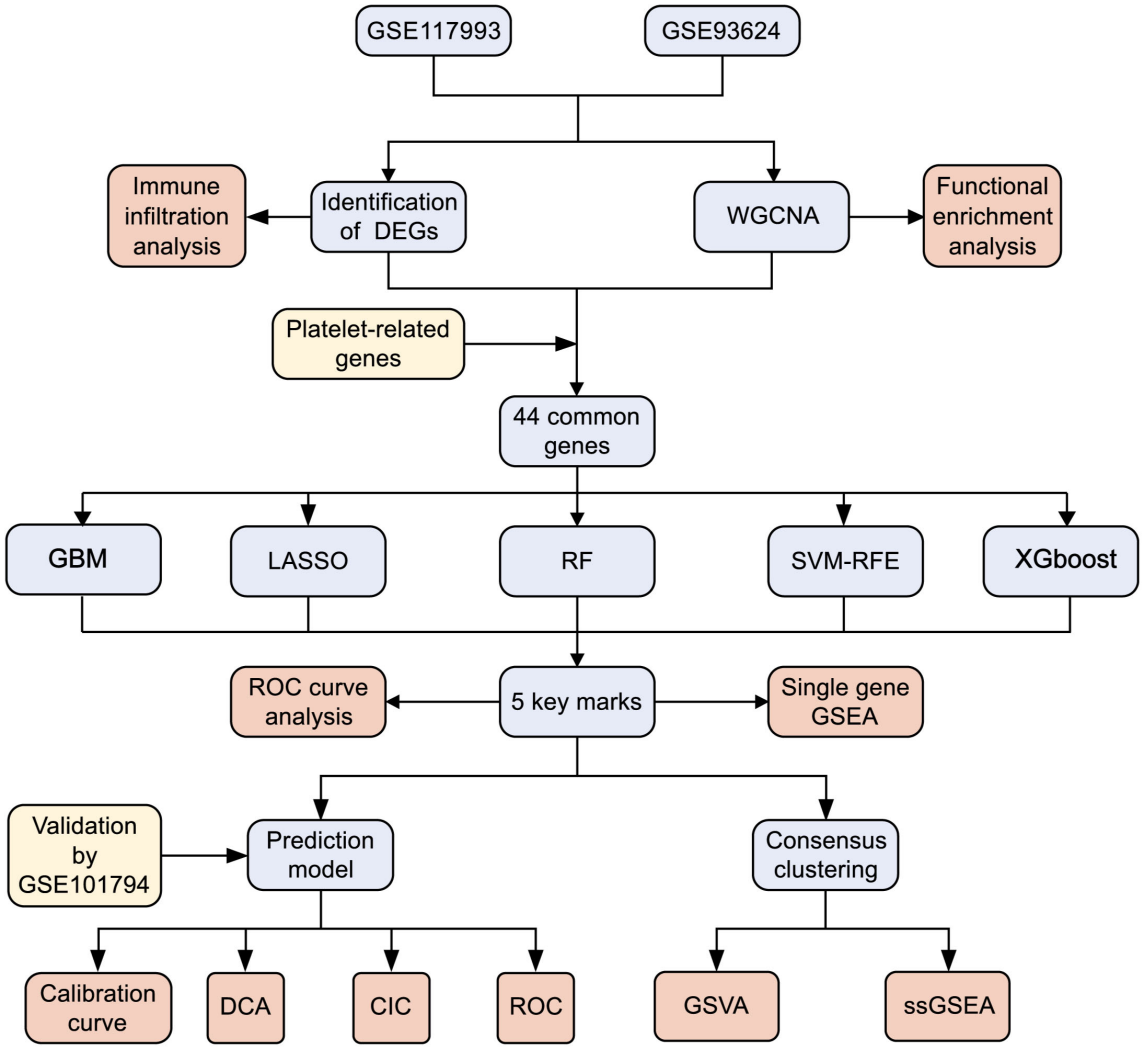
Flowchart for research. DEGs, differentially expressed genes; WGCNA,Weighted gene co-expression network analysis; GBM, Gradient boosting machine; LASSO, Least absolute shrinkage and selection operator; RF, random forest; SVM-RFE, support vector machine-recursive feature elimination; XGBoost, Extreme Gradient boosting; ROC, receiver operating characteristic curve; GSEA, gene set enrichment analysis; DCA, decision curve analysis; CIC, clinical impact curves; GSVA, gene set variation analysis.

Although the precise pathogenic mechanisms of PCD remain enigmatic, platelets have garnered considerable attention as potential players in the genesis of autoimmune-related conditions. Our study aims to contribute to the burgeoning body of research by investigating the regulation of platelet-related genes (PRGs) in PCD through an integrated bioinformatics approach. We also endeavor to devise diagnostic models and molecular subtypes grounded in platelet-associated pathways. Our findings hold the promise of illuminating the pathogenesis and prognostic diversity of PCD. Additionally, the identification of fresh biomarkers holds the potential to facilitate early diagnosis, tailored treatment strategies, and risk stratification for PCD in the future. The schematic depiction of our study’s methodology is outlined in **Figure 1**.

## Methods

### Dataset acquisition and processing

In this study, we obtained three datasets from the Gene Expression Omnibus (GEO) database (https://www.ncbi.nlm.nih.gov/geo/) to support subsequent analysis: GSE117993 (16), GSE93624 (17), and GSE101794 (18). The GSE117993 dataset comprises transcriptome data from 55 normal individuals and 92 patients with PCD. Similarly, GSE93624 contains transcriptome data from 35 normal individuals and 210 PCD patients. Both GSE117993 and GSE93624 were employed as training sets within this study. Additionally, the GSE101794 dataset includes 304 ileal biopsy samples, with 50 sourced from individuals with normal conditions and 254 obtained from PCD patients. This dataset serves as the validation set. Detailed specifics regarding these three datasets can be found in **Table 1** and **Supplementary Table 1**. Subsequently, we integrated the two training datasets using the “limma” package in R software (version 4.2.2) (19). Notably, when multiple probes from distinct platforms identified identical genes, we computed the average value to represent the expression level. We then employed the “sva” packages to normalize and correct for batch effects in the training set (20). Additionally, for the acquisition of platelet-related genes (PRGs), we utilized a gene set consisting of 300 PRGs identified in a previous similar study (11).

**Table 1:** Characteristics of the microarray datasets for PCD.

GEO ID	Platform	Year	PCD	Control	Biopsy Tissue Source
Training set:					
GSE117993	GPL16791	2018	106 PCD samples	55 controls	rectal
GSE93624	GPL11154	2017	210 PCD samples	35 controls	ileum
Validation set:					
GSE101794	GPL11154	2018	254 PCD samples	50 controls	ileum

GEO, Gene Expression Omnibus; PCD, PCD.

### Identification of differentially expressed genes

Differential gene analysis was conducted between the samples using the “limma” package on the normalized dataset. We applied significance criteria with adjusted p-values < 0.05 and |log2 fold change (FC)| > 0.3. Subsequently, the differentially expressed genes (DEGs) that met these criteria were visualized using the volcano plot. Additionally, the heat map was generated to display the top 20 up-regulated DEGs and the top 20 down-regulated DEGs.

### Immune infiltration analysis

The single-sample gene enrichment analysis (ssGSEA) algorithm characterizes the state of a cell by assessing the activity levels of specific biological processes and pathway pairs. In this study, we employed the ssGSEA algorithm, relying on the “GSVA” package (21), to assess the relative infiltration abundance and correlation among immune cells in both the PCD and normal group samples. Subsequently, we visualized the results using the “gglot2” package (https://sourceforge.net/projects/ggplot2.mirror/).

### Identification of platelet-related signature genes based on weighted gene co-expression network analysis (WGCNA)

To probe the potential regulatory associations between genes, we used the “WGCNA” package of the R software to construct gene co-expression networks (22). First, in order to ensure the reliability of the constructed network results, the normal-value samples were screened with mean fragments per kilobase million (FPKM) > 0.5 as the filtering criterion. Second, cluster analysis was performed using the “flashClust” toolkit, retaining the samples that best fit in the cluster under a specified threshold. Third, use the “pickSoftThreshold” function to select the soft threshold of the optimal weighting coefficient β value to establish a scale-free network, thereby transforming the similarity matrix into an adjacency matrix. Fourth, convert the adjacency matrix into a topological overlap matrix (TOM) and calculate the corresponding dissimilarity (1-TOM). Fifth, the module clipping height was set to 0.3 and the minimum number of modules was set to 100, and then the dynamic tree cutting method was used to identify modules from the hierarchical clustering tree. In addition, we calculated the module membership (MM) and gene significance (GS) for the modules correlated to the clinical attributes. Finally, the module most closely related to PCD was screened, and the genes in this module were intersected with the DEGs and PRGs for further analysis.

### Functional enrichment analysis

To investigate the underlying biological mechanisms involved in the development of PCD, we conducted gene ontology (GO) and Kyoto Encyclopedia of Genes and Genomes (KEGG) enrichment analyses on key module genes, respectively. All analysis were performed by the “clusterProfiler” package of the R software (23), and adjusted q-values < 0.05 were set as significant enrichment thresholds. Subsequently, we obtained candidate diagnostic genes for further analysis by intersecting the key modular genes with DEGs and PRGs.

### Feature genes selection for PCD via machine learning

To identify key markers for PCD, we applied five machine learning algorithms: Support Vector Machine Recursive Feature Elimination (SVM-RFE), Least Absolute Shrinkage and Selection Operator Logistic Regression (LASSO), Gradient Boosting Machine (GBM), Extreme Gradient boosting (XGBoost) and Random Forest. These algorithms were implemented using specific R packages: “e1071” for Support Vector Machine Recursive Feature Elimination (available at https://github.com/johncolby/SVM-RFE), “glmnet” (24) for the LASSO logistic regression, “xgboost” for the XGBoost, (available at https://cran.r-project.org/web/packages/xgboost/) “caret” for the GBM (available at https://cran.r-project.org/web/packages/caret/), and “randomForest” (available at https://cran.r-project.org/web/packages/randomForest/) for Random Forest. It’s noteworthy that in this study, both the LASSO regression and SVM-RFE algorithms were evaluated using 10-fold cross-validation to estimate their prediction performance. Additionally, for the Random Forest (RF) algorithm, genes with a relative importance greater than 2.5 were considered key markers. For the “XGBoost” and “GBM” algorithms, we selected the top ten genes in terms of importance for the screening of candidate marker genes. The signature genes were determined by identifying the intersection of genes screened by these five algorithms. To visually represent the relationships among the signature genes, we utilized the “circlize” package to create a heatmap of gene correlations (25). These maps illustrate the interplay between the roles of these genes. Additionally, we generated heat maps based on Spearman correlation analysis to depict the relationships between the signature genes and 23 immune cell types.

### Gene Set Enrichment Analysis (GSEA) for single diagnostic marker

To thoroughly investigate the potential connections between key diagnostic genes and signaling pathways, we conducted a comparative analysis of biosignaling pathways between the disease and control groups. Subsequently, we generated ridge maps utilizing the “clusterProfiler” package to visually represent the top 10 enrichment results for each gene.

### Establishment and validation of the nomogram for PCD

We developed a clinical prediction model for PCD based on these signature genes, utilizing the “gglot2” package. The calibration curve was used to evaluate the nomogram model’s predictive accuracy. Then, we performed decision curve analysis and clinical impact curve analysis to estimate the nomogram model’s clinical utility. Furthermore, we assessed and validated the predictive performance of each signature gene in both the training and validation sets using receiver operating characteristic (ROC) curves. A larger area under the curve (AUC) in the ROC analysis indicated better predictive performance of the model. Finally, we use the dataset GSE101794 as a validation set to test whether the prediction model is generalizable.

### Identification of platelet-associated molecular subtypes of PCD by consensus cluster analysis

Consensus clustering is an unsupervised algorithm designed to identify and cluster individual samples within a dataset, facilitating the distinction of various subtypes. In this study, we applied consensus clustering to differentiate between distinct subtypes of platelet-related PCD molecules. This differentiation was based on the feature genes identified by five machine learning algorithms, and we utilized the “ConsensusClusterPlus” package for this purpose (26). The optimal number of clusters was determined through the analysis of consensus cumulative distribution function (CDF) plots, principal component analysis (PCA), consensus matrix plots, relative changes in the area under the CDF curve, and tracking plots. To explore variances in platelet-associated pathways across different molecular subtypes, we employed the gene set variation analysis (GSVA) algorithm and visualized the results. Additionally, for the analysis of discrepancies in immune cell infiltration status among subtypes, we conducted the ssGSEA algorithm using R, followed by the Wilcoxon test to assess differences in the immune microenvironment.

## Results

### Data processing and identification of DEGs

**Figure 2:**
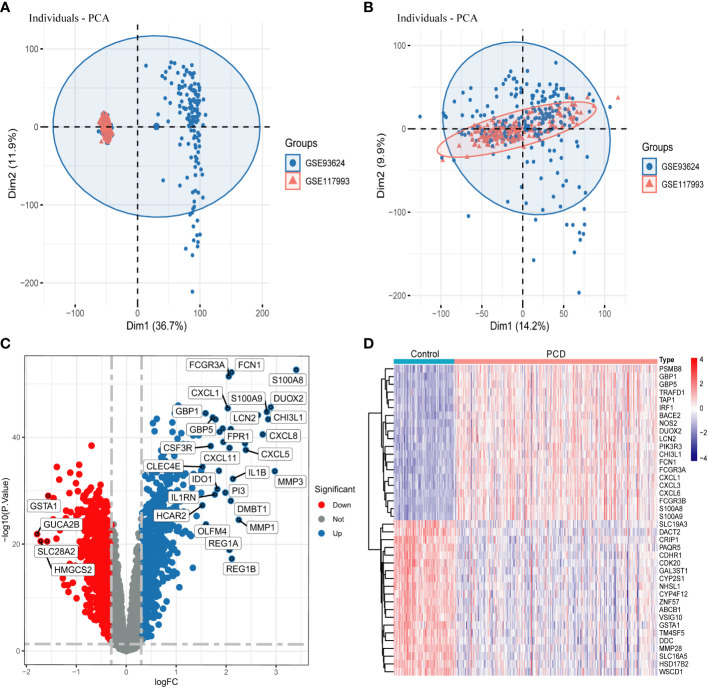
Data processing and screening for DEGs. **(A, B)** PCA plots showing the difference in expression profiles of the combined GSE93624 and GSE117993 dataset before and after the de-batch effect. **(C)** Volcano plot of DEGs in the combined dataset. **(D)** Heatmap of DEGs in the combined dataset. DEGs, differentially expressed genes.

The training set consists of GSE117993 and GSE93624 and contains a total of 302 PCD patients and 90 normal patient samples. After the ComBat algorithm to remove the batch effect, all the samples in the training set were centered and well-distributed, indicating the high quality and cross-comparability of this microarray dataset (**Figures 2A, B**). Subsequent differential analysis eventually screened out 1643 genes, including 887 up-regulated genes and 756 down-regulated genes (**Figure 2C** and **Supplementary Table 1**). The heatmap demonstrated the top 20 up- and down-regulated DEGs between PCD patients and the control group (**Figure 2D**).

### Immune infiltration analysis

**Figure 3:**
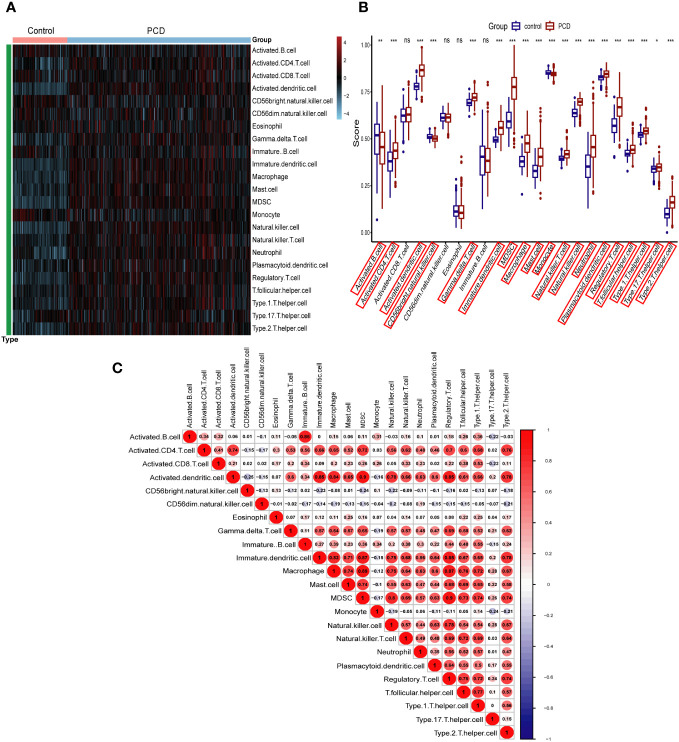
Immune cell infiltration analysis in combined datasets. **(A)** Heatmap of all PCD patient and control samples with 23 immune cells. **(B)** Boxplot depicting the level of immune cell infiltration in PCD patients and controls. **(C)** Heatmap depicting the correlations between distinct immune cell compositions. PCD, pediatric Crohn’s disease; *p < 0.05, **p < 0.01, ***p < 0.001; ns, no significance.

In this study, we used the ssGSEA algorithm to compare the levels of immune cell infiltration between the PCD patients and the normal group in the combined dataset. **Figure 3A** depicts the difference in expression of 23 immune cells in PCD samples and normal samples. Box plots were used to show the differences in immune cell infiltration between the PCD and control groups (**Figure 3B**). The results showed that cells such as activated CD4 T cells, activated B cells, macrophages, and neutrophils were significantly higher in the PCD patient group compared to the control group. In addition, we investigated the relationship between immune cells. As illustrated by the correlation heatmap in **Figure 3C**, macrophages, immature dendritic cells, mast cells, activated dendritic cells, and type 2 T helper cells exhibited strong correlations with the majority of other immune cell types.

### Identification of platelet-related signature genes based on WGCNA

**Figure 4:**
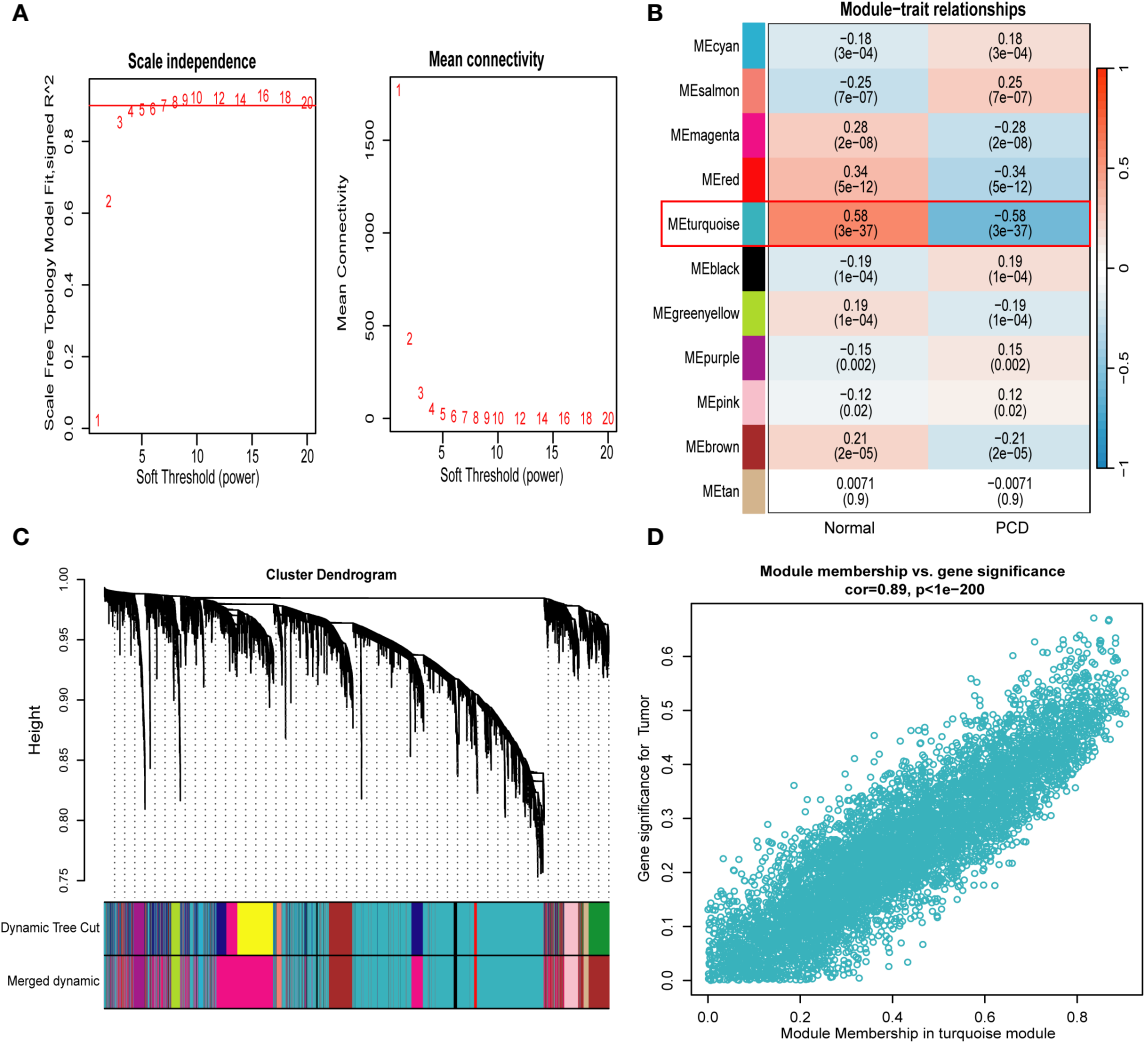
Weighted gene co-expression network analysis. **(A)** Identification of soft−threshold powers. R^2^ = 0.90. **(B)** Relationships between modules and traits in PCD and control. The numbers in each cell represent the correlation coefficient and p-value. **(C)** Cluster tree for all genes in the combined dataset. **(D)** Scatterplot of genes in the turquoise module with the strongest correlation to PCD. PCD, pediatric Crohn’s disease.

We used WGCNA to filter the set of genes that are more consistent with PCD and selected the optimal soft threshold based on the results of the “pickSoftThreshold” function of the “WGCNA” package. In addition, in order to make the co-expression network conform to the scale-free principle, β=7 (scale-free R^2^ = 0.9) was chosen to construct the gene co-expression network in this study (**Figure 4A**). Finally, we converted the similarity matrix to TOM. Next, based on TOM, we used the previously mentioned criteria to merge the modules that were closer together, and finally obtained a total of 11 modules (**Figures 4B, C** and **Supplementary Table 2**). Finally, based on the results of the module-trait association analysis, we selected the turquoise module with the strongest association with PCD (5997 genes in total). In addition, a substantial positive correlation was identified between module membership and gene significance in the turquoise module for PCD samples (r = 0.89, p = 1e-200), as illustrated in **Figure 4D**.

### Functional enrichment analysis

**Figure 5:**
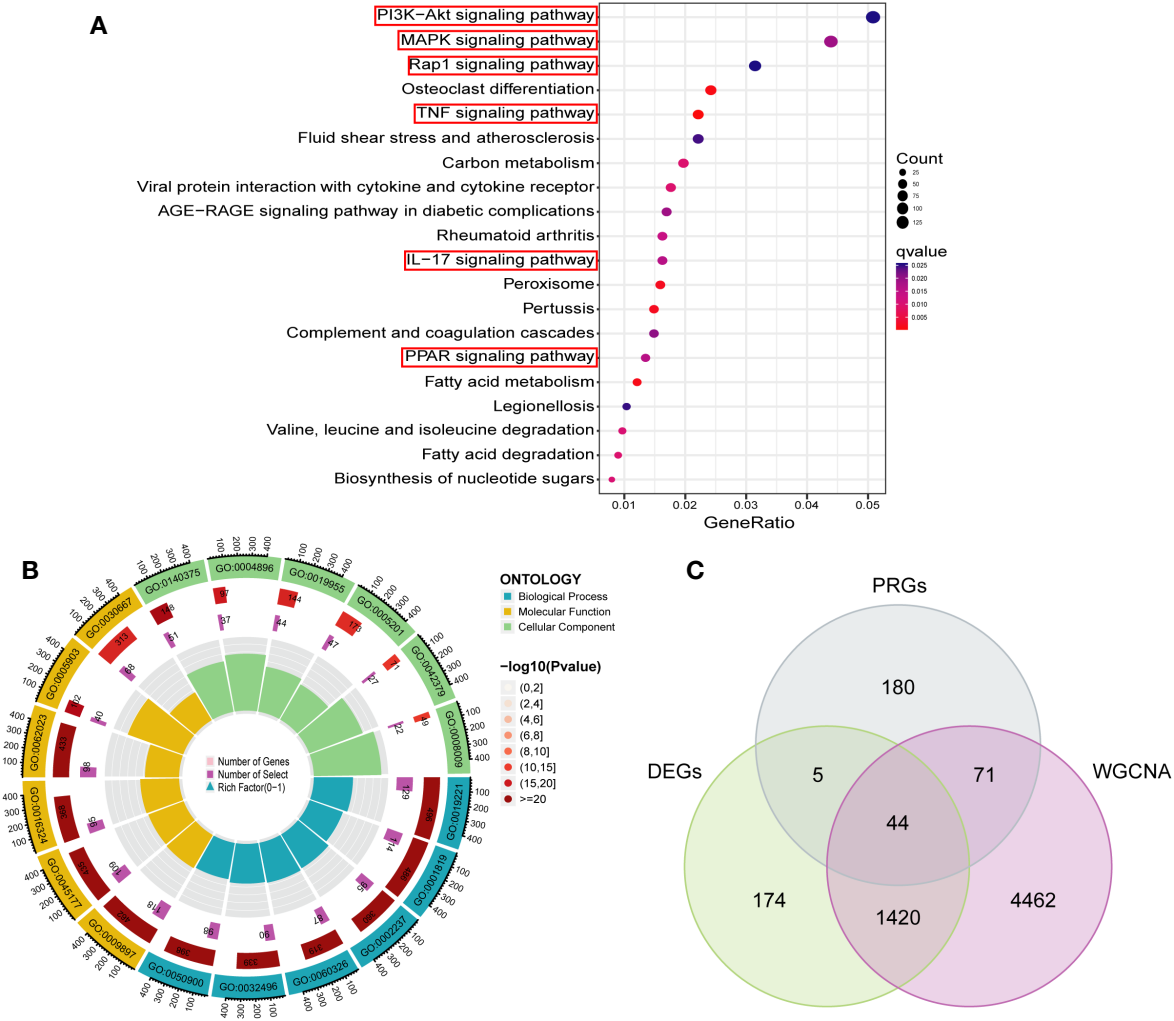
Functional enrichment analysis of key module genes and identification of candidate diagnostic genes. **(A)** The bubble plot showing the most enriched KEGG pathways of key module. **(B)** Gene Ontology analysis of key module. **(C)** Candidate diagnostic genes were obtained by overlapping key modules, DEGs and PRGs. DEGs, differentially expressed genes; PRGs, platelet-related genes; KEGG, kyoto encyclopedia of genes and genome; BP, biological process; CC, cellular component; MF, molecular function.

In order to investigate the biological functions and pathways associated with the turquoise modular gene set, which exhibited the strongest association with PCD as identified through WGCNA, in the context of PCD development, we conducted GO and KEGG analyses on this gene set. This analysis yielded a total of 37 significantly enriched signaling pathways and 1661 biological processes that displayed significant associations (refer to **Supplementary Table 3**). As depicted in **Figure 5A**, these genes were predominantly enriched in signaling pathways closely linked to immune and inflammatory responses, including but not limited to the PI3K-Akt, MAPK, TNF, IL-17, and Rap 1 pathways. The GO enrichment analysis indicated that the majority of these genes were primarily involved in cytokine-mediated signaling pathways, positive regulation of cytokine production, responses to molecules of bacterial origin, cell chemotaxis, and reactions to lipopolysaccharides and polysaccharides, among other biological processes, as illustrated in **Figure 5B**. Furthermore, we employed a Venn diagram, as depicted in **Figure 5C**, to identify an intersection between the key module gene set and DEGs as well as PRGs. This intersection yielded a set of 44 candidate diagnostic genes for subsequent analysis.

### Screening signature genes by machine learning

**Figure 6:**
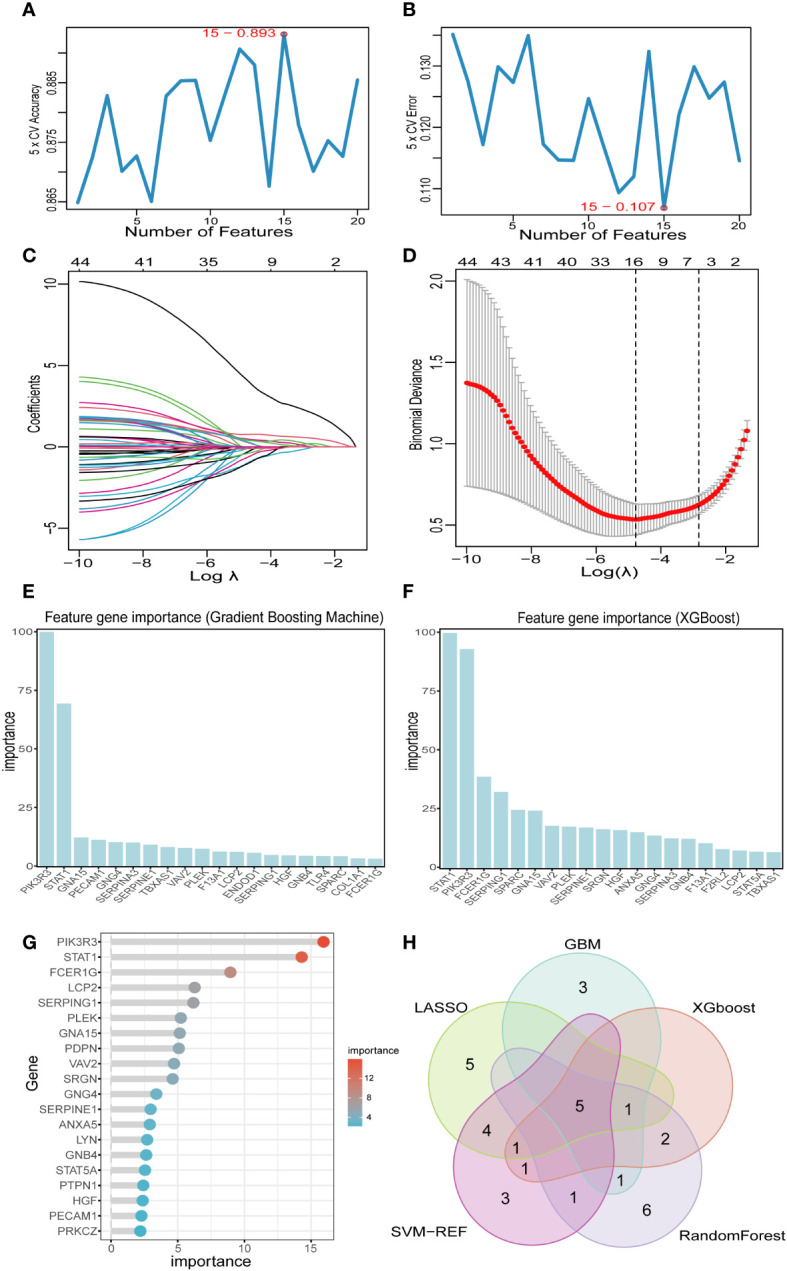
Screening key markers by machine learning. **(A, B)** Fifteen feature genes were screened by SVM-RFE algorithm. **(C, D)** Sixteen feature genes were screened by LASSO algorithm. **(E)** The top 20 genes in terms of importance as filtered by the GBM algorithm. **(F)** The top 20 genes in terms of importance as filtered by the XGBoost algorithm. **(G)** Sixteen feature genes were screened by RF algorithm. **(H)** Five key markers were screened by Venn diagram of five algorithms. LASSO, least absolute shrinkage and selection operator; SVM-RFE, support vector machine-recursive feature elimination; GBM, Gradient Boosting Machine; XGBoost, Extreme Gradient boosting; RF, random forest.

Based on the 44 key genes screened in the previous step, we used five machine learning algorithms to further identify potential platelet-related biomarkers for PCD. In the SVM-RFE algorithm, we finally identified 15 featured genes (**Figures 6A, B**). In the LASSO algorithm, we screened 16 feature genes (**Figures 6C, D**). In the “XGBoost” and “GBM” algorithms, we filtered out the top 10 feature genes in terms of importance (**Figures 6E, F**).And in the RF algorithm, we finally screened a total of 16 feature genes with relative importance greater than 2.5 (**Figure 6G** and **Supplementary Figure 1**). Subsequently, the Venn diagram showed that there were five identical genes among the five machine learning algorithms: PIK3R3, STAT1, PLEK, GNA15, and SERPINE1 (**Figure 6H**).

### The association of key markers with immune cells and GSEA

**Figure 7:**
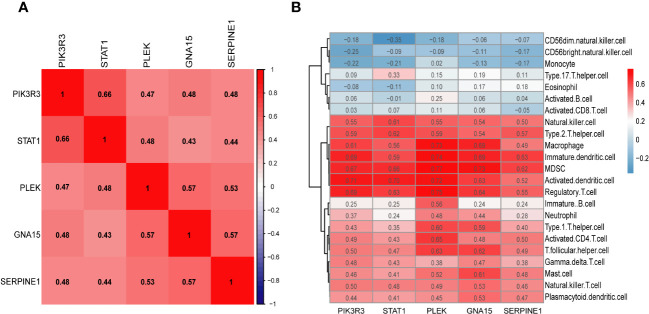
**(A)** The heatmap shows the relationship between the key markers. **(B)** Correlation heatmap depicting the relationship between immune cell infiltration and key markers.

**Figure 8:**
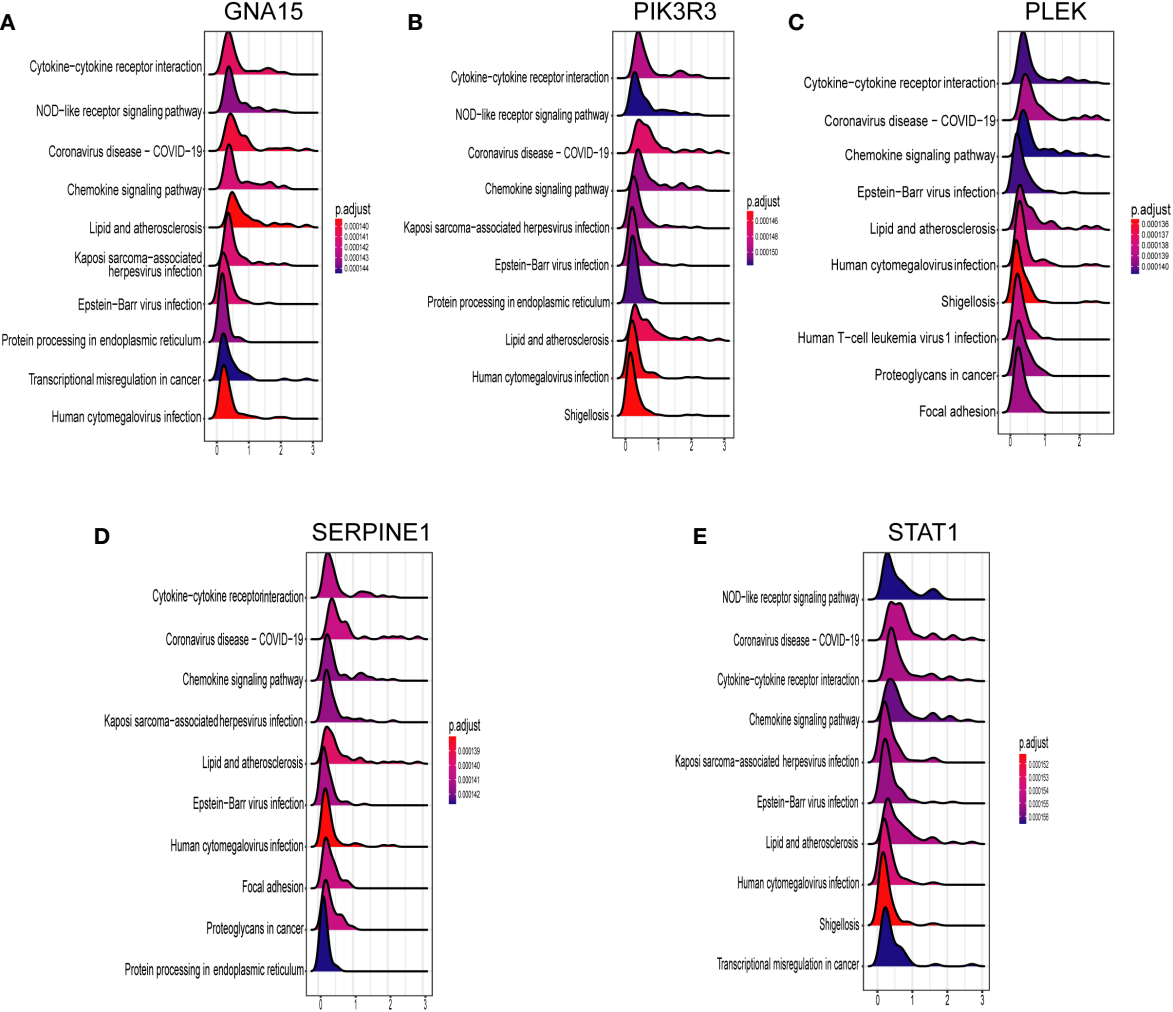
GSEA for the single mark. **(A)** GNA15. **(B)** PIK3R3. **(C)** PLEK. **(D)** SERPINNE1. **(E)** STAT1.

We conducted Spearman correlation analysis to elucidate the interrelationships among the diagnostic genes and their potential association with immune cell infiltration, thereby enhancing our understanding of the functional role of these pivotal markers in immune infiltration. The correlation analysis revealed a robust positive correlation among all five diagnostic genes (all exceeding 0.4), as illustrated in **Figure 7A**. Furthermore, the associations between these five diagnostic genes and a majority of the immune cells exhibited a highly consistent pattern. Specifically, all five diagnostic markers displayed varying degrees of negative correlation with activated B cells, CD56 bright natural killer cells, CD56dim natural killer cells, monocytes, eosinophils, T-helper cells type 17, and activated CD8 T cells. Additionally, there existed an overall strong positive correlation between the remaining 16 immune cells and these five diagnostic genes, as depicted in **Figure 7B**. As demonstrated in **Figures 8A–E**, GNA15, PIK3R3, PLEK, SERPINE1, and STAT1 exhibited significant enrichment in inflammatory and immune-related pathways, such as cytokine-cytokine receptor interaction, NOD-like receptor signaling pathway, and chemokine signaling pathway.

### Establishment and validation of the nomogram for PCD

**Figure 9:**
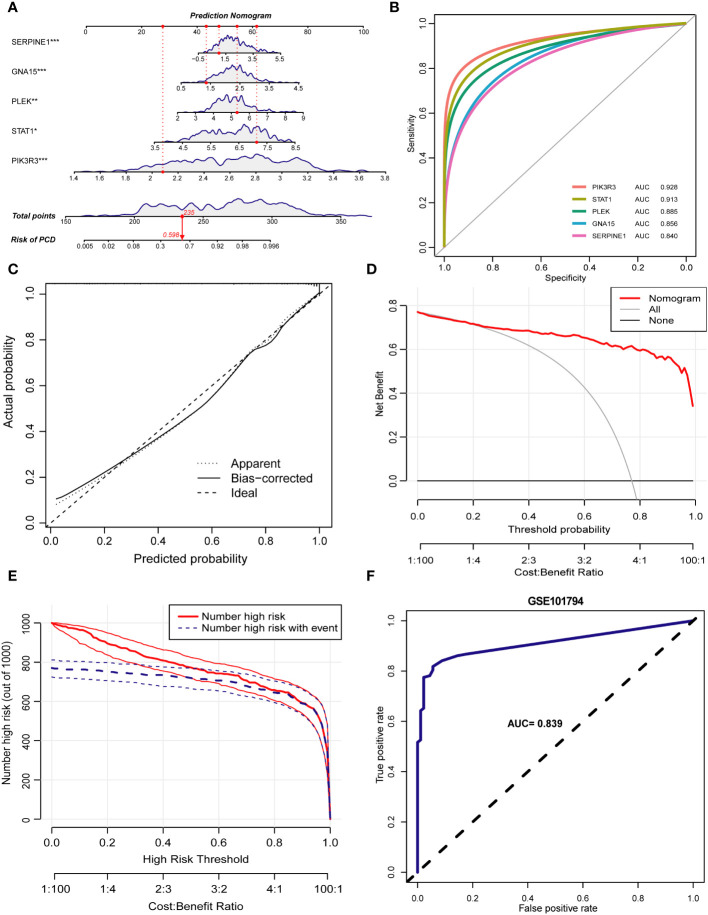
Construction of the prediction model. **(A)** Nomogram of PCD patients based on 5 key markers. **(B)** ROC curve of key markers in PCD diagnosis. **(C)** The calibration curve demonstrates nomogram’s excellent predictive performance. **(D)** The results of the decision curve analysis showed a good net clinical benefit rate for the model. **(E)** Assessing the clinical impact of the nomogram by clinical impact curves. **(F)** The ROC curve demonstrates the predictive power of the predictive model in the validation set GSE101794. *p < 0.05, **p < 0.01, ***p < 0.001.

Inspired by the results of previous studies, we constructed a clinical risk prediction model for PCD using the five signature genes that were finally screened (**Figure 9A**). In addition, the ROC-AUCs of the five characterized genes were higher than 0.8, indicating that the model has good discriminative performance (**Figure 9B**). The calibration curve of the model showed that the nomogram performed close to ideal with excellent predictive consistency (**Figure 9C**). As shown in **Figure 9D**, there is a high net clinical benefit for clinicians and patients using this model. The clinical impact curve further confirms the high clinical efficacy of this predictive model (**Figure 9E**). Finally, we further validated the predictive ability of the model in an external dataset GSE101794. In the validation set, the AUC of the model is 0.839, indicating that the model has some stability and generalizability (**Figure 9F**).

### Identification of platelet-associated molecular subtypes of PCD by consensus cluster analysis

**Figure 10:**
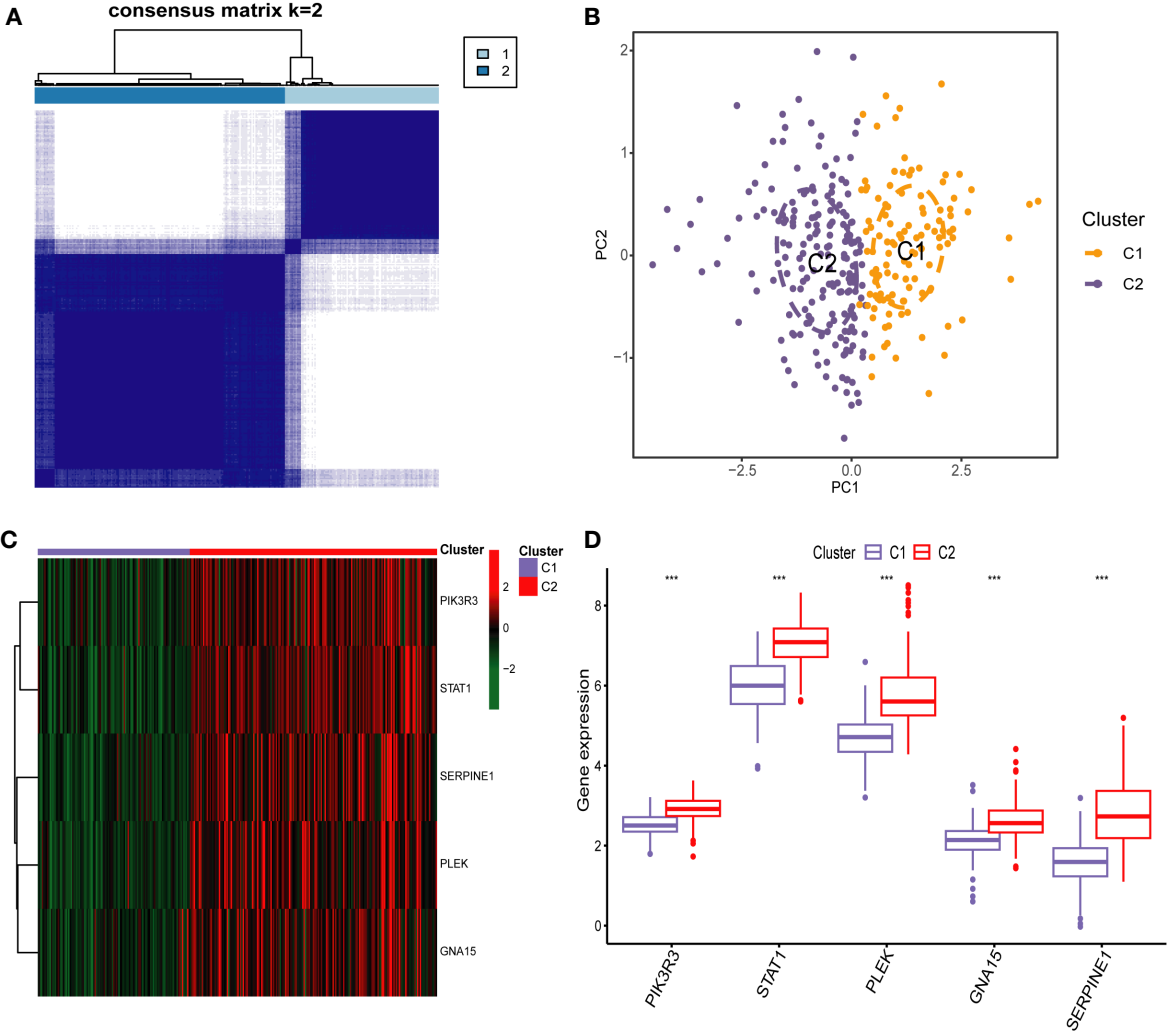
Identification of Platelet-Related Molecular Subtypes of PCD by Consensus Cluster anaylsis. **(A)** Consensus matrix heatmap for k = 2. **(B)** Sample distributions of the two subtypes were visualized by principal component analysis. Heatmap **(C)** and boxplot **(D)** demonstrating the expression differences of the five key markers in different subtypes. *** p < 0.001.

**Figure 11:**
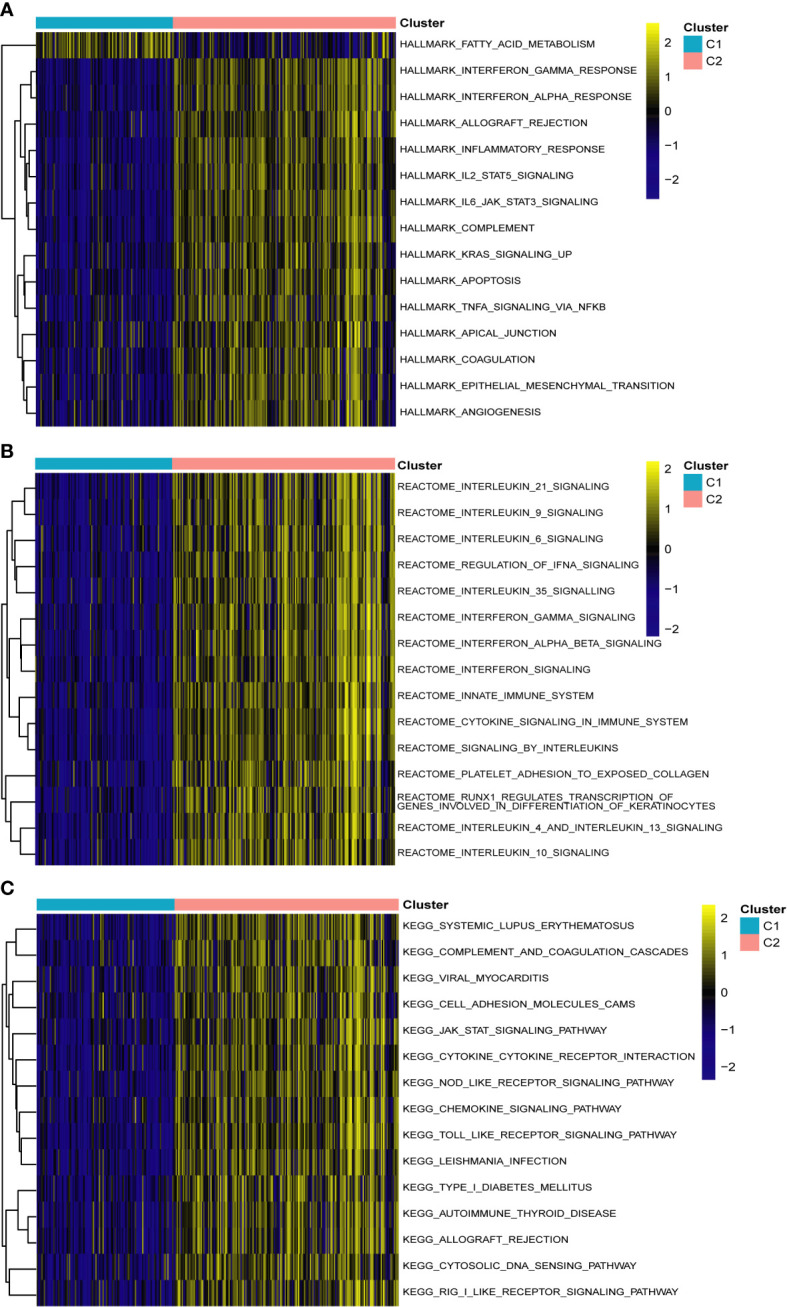
Gene set variation analysis between two different subtypes of PCD. **(A)** Enriched pathways based on the hallmark pathway. **(B)** Enriched pathways based on the reactome pathway. **(C)** Enriched pathways based on the KEGG pathway.

**Figure 12:**
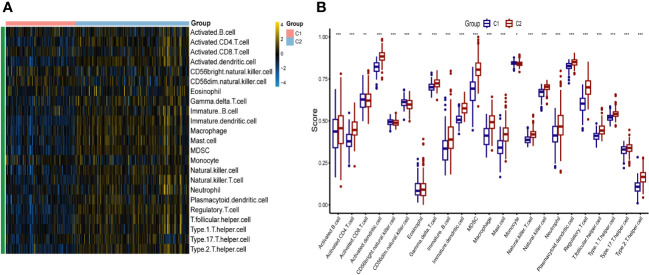
The immune landscape of two different platelet subtypes of PCD. Heatmap **(A)** and boxplot **(B)** demonstrating the differences in immune cell infiltration between different subtypes. *p < 0.05, **p < 0.01, ***p < 0.001.

To identify different PCD molecular subtypes, we analyzed the expression profiles of five characteristic genes in all PCD samples in the training set based on a consensus clustering approach. The consensus matrix plot, CDF plot, and the area under the CDF curve change plot finally identified K=2 as the optimal number of subtypes (**Figure 10A** and **Supplementary Figures 2A–C**). We named the two platelet molecular subtypes as C1 (n=115) and C2 (n=187) (**Supplementary Table 4**). Principal component analysis revealed significant heterogeneity in gene expression patterns between the two subtypes (**Figure 10B**). Subsequently, we also highlighted the expression pattern of PDEGs in the two subtypes with heatmap and boxplot (**Figures 10C, D**). It was evident that the expression of the five key marks was significantly upregulated in the C2 cluster. In order to delve further into the potential pathogenic mechanisms of each subtype and evaluate disparities in enriched pathways between them, we conducted GSVA for the two subtypes. Our findings revealed notable distinctions. For instance, the C2 cluster exhibited significantly greater enrichment compared to the C1 cluster across the majority of the HALLMARK pathways, encompassing responses to interferon gamma and interferon alpha, xenograft rejection, and inflammatory response. Notably, the fatty acid metabolism pathway displayed heightened enrichment in C1 clusters as opposed to C2 clusters (**Figure 11A**). Likewise, C2 clusters demonstrated significantly greater enrichment than C1 clusters in pathways involving interleukin 6, 9, 21, 35, and cytokine signaling within the immune system (**Figure 11B**). Additionally, our analysis based on the KEGG database revealed a significant enrichment in pathways like systemic lupus erythematosus, complement coagulation cascade reaction, and cell adhesion molecules for the C2 cluster (**Figure 11C**). It is imperative to note that in subsequent immune cell infiltration analysis, we observed a substantially higher abundance of immune infiltration in the C2 cluster across all 23 immune cell types, aligning consistently with the findings from the GSVA (**Figures 12A, B**).

## Discussion

PCD is a refractory disease that has a major impact on all aspects of a child’s life. In terms of nutrition, chronic inflammation and malabsorption can lead to nutritional deficiencies and growth disorders that affect children’s development and overall health (27). On a psychological level, living with a chronic disease can take a toll on a child’s mental and emotional health, potentially leading to anxiety, depression, and decreased quality of life (28). In terms of school and social activities, frequent symptoms and medical appointments can disrupt a child’s educational and social life, requiring support from healthcare providers and educators (29). In addition, the vast majority of children with PCD are also at risk for long-term complications, and if not managed properly, PCD can lead to serious complications, including an increased risk of strictures, fistulas, and colorectal cancer (30). This is despite the fact that research into the development of PCD has achieved some milestones in recent years (31). However, issues such as delayed diagnosis, growth and developmental problems, drug safety, psychosocial support, and access to health care remain. Therefore, accurate differentiation of PCD clusters at the molecular level will improve our understanding of PCD heterogeneity and is essential to guiding individualized treatment of PCD. And platelet-related genes have demonstrated excellent diagnostic performance and molecular subtype discrimination in previous studies. However, whether platelets are associated with the development of PCD and the underlying molecular mechanisms of platelets in PCD remain unclear. Therefore, the exploration of new platelet-related diagnostic markers and risk stratification for children with PCD remains an urgent need.

In this study, we identified 887 up-regulated and 756 down-regulated DEGs from 302 PCD cases and 90 normal control samples using data from the GSE93624 and GSE117993 datasets. Employing WGCNA, we isolated 5997 pivotal modular genes closely associated with PCD. Subsequent GO enrichment analysis revealed that these key module genes were primarily enriched in biological processes, including the positive regulation of cytokine production, the negative regulation of immune system processes, and leukocyte migration. Positive regulation of cytokine production means increasing the expression or activity of cytokines, which are important for both normal and abnormal cell-mediated immune responses and intestinal inflammation. Cytokines play a central role in CD as they can either promote or suppress gut inflammation (32, 33). Leukocyte migration, also known as leukocyte transport, plays a pivotal role in the pathogenesis of IBD (34). In IBD, there is a chronic inflammatory cell infiltrate in the intestinal mucosa, and immune cells are transported to the gastrointestinal tract through specific mechanisms. Integrins, cell surface receptors, bind cell adhesion molecules, facilitating leukocyte homing and retention. Therapies targeting leukocyte trafficking may play a crucial role in achieving stratified precision medication administration in the care of IBD (35). However, it is important to note that patients may exhibit varying responses to these treatments, underscoring the need for additional mechanistic studies in clinical trials. Furthermore, KEGG enrichment analysis revealed that the development of PCD is primarily associated with signaling pathways such as PI3K-Akt, MAPK, and Rap 1. The PI3K-Akt, MAPK, and Rap 1 pathways are pivotal in various cellular processes, including cell growth, proliferation, differentiation, and survival (36). Aberrant activation of these pathways has been linked to various diseases, including cancer (37). While specific information regarding the relationship between these pathways and IBD is lacking, it is established that inflammation and immune responses play a significant role in IBD. These pathways may potentially influence these processes. For example, the PI3K-Akt pathway is known to regulate immune and inflammatory responses (38), and the MAPK pathway also plays a role in proinflammatory cytokine production (39). As for the Rap 1 pathway, although its role in IBD remains to be fully elucidated, Rap 1 is involved in a variety of cellular processes, including cell adhesion and cell-cell interactions, which could have implications for the pathogenesis of IBD (37). In a subsequent immune cell infiltration analysis, we found that 19 types of immune cells, including activated B cells, activated CD4 T cells, and monocytes, had higher infiltration abundance in PCD patients. Previous studies have found that in patients with Crohn’s disease, B cells show signs of chronic stimulation and are localized to granulomatous tissue. These cells also exhibit increased molecular maturation of IgA and IgG1 (40). In addition, there are studies that have identified distinct subpopulations of B cells that are highly infiltrated in the lamina propria of patients with Crohn’s disease. These cells express genes related to antigen presentation. This subset of B cells is thought to play a potential promoting role in the pathogenesis of Crohn’s disease (41). In previous studies, CD4 T cells were found to play a key role in the pathogenesis of Crohn’s disease (42). These cells can differentiate into regulatory and effector T cells, such as Th1, Th2, Th17, follicular helper T cells (Tfh), and regulatory T-cells (Tregs), depending on the cytokine milieu. In particular, CD4+ tissue-resident memory T (T_RM) cells have been found to be expanded in Crohn’s disease and are the major source of mucosal tumor necrosis factor α (TNFα), a principal mediator of intestinal injury. There is a unique population of TNFα + IL-17A + CD4 + T_RM cells in Crohn’s disease which are largely absent in controls (43). In the context of Crohn’s disease, recent developments suggest that there is an impaired monocyte function initiating the disease and an overactivation of monocytes and adaptive immunity maintaining the disease (44). Monocytes and monocyte-derived macrophages have been found to be crucial players in the chronic inflammation seen in Crohn’s disease patients (45).

We performed an intersection analysis involving DEGs, key modular genes identified through WGCNA, and PRGs. This resulted in the identification of 44 potential diagnostic markers. Subsequently, we employed five machine learning algorithms to further screen for the top five diagnostic biomarkers associated with PCD: PIK3R3, STAT1, PLEK, GNA15, and SERPINE1, which exhibited the strongest diagnostic capabilities. PIK3R3 serves as the regulatory subunit of phosphatidylinositol kinase, a crucial signaling pathway implicated in cell growth, proliferation, and immune response. This kinase plays a pivotal role in various essential mediators of cellular processes, including inflammation and immune regulation. In a study conducted by Wang et al., they observed that the overexpression of PIK3R3, primarily dependent on SNAI2, triggers a significant intestinal epithelial-mesenchymal transition (EMT). Furthermore, the downregulation of PIK3R3 reversed this process, potentially leading to increased invasion and metastasis of colorectal cancer cells (46). Signal transduction and activator of transcription 1 (STAT1) plays a multifaceted role in the pathogenesis of Crohn’s disease, an IBD. Research indicates that STAT1 is involved in the epigenetic regulation of two key genes, Lymphocyte Cytosolic Protein 2 (LCP2) and TNF-α‐inducible protein 2 (TNFAIP2), by recruiting EP300, thereby contributing to the development of IBD. Specifically, phosphorylated STAT1 (p-STAT1) binds to the enhancer loci of these two genes, where H3K27ac enrichment is notable, leading to subsequent EP300 binding and the regulation of gene expression. This process promotes the expression of TNFAIP2 and LCP2 by augmenting the H3K27ac enrichment on their enhancers, ultimately contributing to the pathogenesis of chronic inflammation (47). Furthermore, a study employing an acute colitis model revealed significant improvements in disease state among STAT1-deficient mice in comparison to wild-type mice. Notably, the induction of highly expressed Ly6c cells in colorectal tissues was notably reduced in STAT1-deficient mice (48). Serpin Peptidase Inhibitor 1 (SERPINE1) has been identified as a potential new disease activity marker in IBD, which includes Crohn’s disease. In a study, it was found that the expression of SERPINE1 differed significantly in healthy subjects compared to IBD patients with active disease. After therapy induction, a remarkable decrease was observed in the mucosal SERPINE1 concentration in responders. Moreover, the study found that serum SERPINE1 correlates with disease activity (p<0,01, cut-off value: 22 mg/ml, sensitivity = 80%, specificity = 60%, accuracy = 74%), whereas no correlation was observed between the mucosal SerpineE1 concentration and the disease activity (p > 0.1, sensitivity = 72%, specificity = 77.8%, accuracy = 73.5%). These findings suggest that SERPINE1 could potentially be used as a marker to monitor disease activity and therapeutic response in IBD, including Crohn’s disease (49). PLEK, also known as Pleckstrin, encodes a protein involved in intracellular signaling and the regulation of cell growth, proliferation, and differentiation. It plays a role in diverse cellular processes, including platelet activation and immune response regulation (50). The study by Chen and Medrano et al. reported that aberrant expression of PLEK may be involved in the pathogenesis of IBD by increasing inflammatory factors, which is consistent with our findings (51, 52). GNA15 is a gene that encodes a subunit of a guanine nucleotide-binding protein (G protein). The G proteins, including GNA15, are known to be involved in signal transduction pathways that regulate immune responses. Dysregulation of G protein signaling could potentially contribute to abnormal immune responses and inflammation, which are key features of Crohn’s disease (53). However, direct evidence linking GNA 15 to Crohn’s disease or IBD is lacking in the current literature.

In clinical practice, accurate diagnosis of PCD is often accompanied by the performance of invasive procedures, which causes great pain to the affected children. In addition, due to the heterogeneity of PCD, the same therapeutic regimen is not effective for all PCD patients. Therefore, the creation of predictive modeling tools at the genetic level to look for potential molecular subtypes of PCD, while imposing some relatively large burdens in terms of testing costs, nevertheless, the new predictive modeling and molecular typing will not only be effective in reducing the suffering of the children, but also provide for improved diagnostic accuracy, as well as risk stratification and grading of patients with PCD. In this study, we developed a novel predictive model based on five biomarkers for predicting the risk of developing PCD. In subsequent internal and external validations, it demonstrated excellent predictive accuracy and good clinical benefit rates. This suggests that such work will enable accurate assessment and management of patients when encountered in clinical practice. In addition, we identified two platelet molecular subtypes of PCD based on these five markers. In a subsequent pathway enrichment analysis, we found that the marker activity of the C2 cluster was significantly higher than that of the C1 cluster in all pathways except the fatty acid metabolism pathway.In addition, immune infiltration analysis revealed significant differences in the degree of infiltration of both subtypes in 23 immune cells. This provides a new theoretical basis for risk stratification and personalized diagnosis and treatment of PCD. However, this study still has some limitations. First, selection and detection bias could not be completely avoided because the data used in this study were all from the same database. Second, despite the completion of a large number of secondary analysis of previous data, large-scale experiments at the clinical, cellular, and molecular levels, as well as prospective studies, are still needed to further validate our conclusions.

## Conclusions

In summary, this study unveils, for the first time, a potential association between PCD and PRGs through the integration of WGCNA with machine learning algorithms. Furthermore, we identified five novel diagnostic markers: PIK3R3, STAT1, PLEK, GNA15, and SERPINE1. The nomogram constructed using these five markers present the prospect of a non-invasive approach to diagnosing PCD. Additionally, we have, for the first time, classified PCD into two distinct molecular subtypes. This breakthrough may offer fresh perspectives for exploring the heterogeneity of clinical presentations and prognosis of PCD. Moreover, it lays a theoretical foundation for early prevention, risk stratification, and personalized diagnosis and treatment of UC in the future.

## Data availability statement

Publicly available datasets were analyzed in this study. This data can be found here: https://www.ncbi.nlm.nih.gov/geo/. The accession numbers can be found in the article.

## Author contributions

DT: Formal Analysis, Methodology, Visualization, Writing – original draft. YH: Data curation, Formal analysis, Writing – original draft. YC: Data curation, Methodology, Visualization, Writing – original draft. CY: Data curation, Formal analysis, Visualization, Writing – original draft. BP: Data curation, Methodology, Validation, Visualization, Writing – original draft. SL: Validation, Writing – review & editing. HL: Supervision, Validation, Writing – review & editing.

## Supplementary material

Supplementary Figure 1 Error rate plot for cisualizing Random Forest algorithm results.

Supplementary Figure 2 Consensus Cluster anaylsis. **(A)** Consensus CDF delta area curves when k = 2-9. **(B)** Relative alterations in the area under CDF curve. **(C)** Tracking plot showing the sample classification when k = 2–9. CDF, cumulative distribution function.

Supplementary Table 1 Differentially expressed genes matrix.

Supplementary Table 2 Module cutting results by Weighted gene co-expression network analysis.

Supplementary Table 3 Results of Gene Ontology and Kyoto Encyclopedia of Genes and Genome analysis.

Supplementary Table 4 Results of consensus cluster anaylsis.
