# Supplementary figures and images for: Identification of platelet-related subtypes and diagnostic markers in pediatric Crohn’s disease based on WGCNA and machine learning
Source: Front Immunol. 2024 Feb 14;15:1323418. doi: 10.3389/fimmu.2024.1323418 (PMC10899512; doi:10.3389/fimmu.2024.1323418)

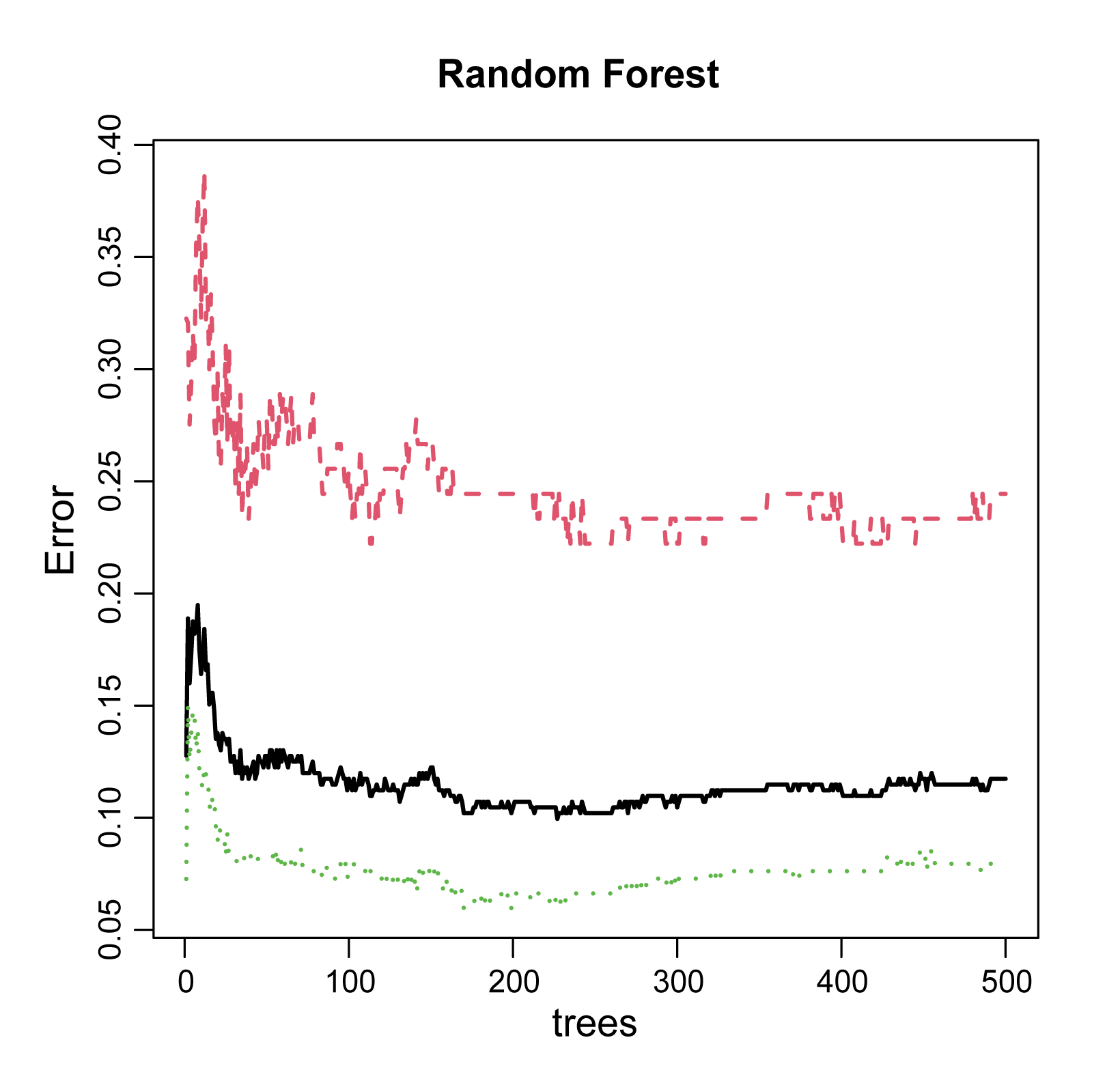

Supplement: Supplementary Figure 1 — Error rate plot for cisualizing Random Forest algorithm results. [file Image_1.tif]

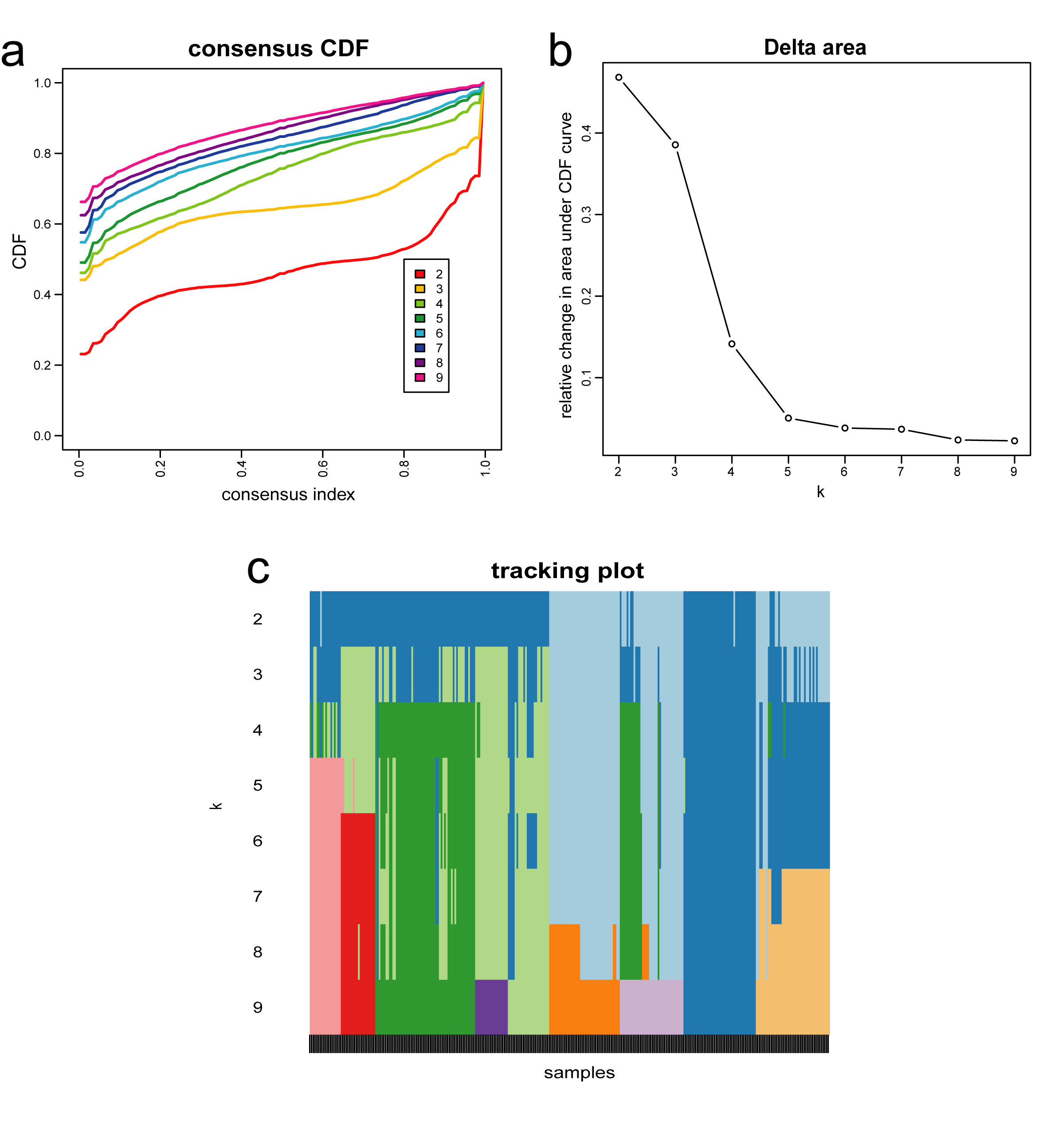

Supplement: Supplementary Figure 2 — Consensus Cluster anaylsis. (A) Consensus CDF delta area curves when k = 2-9. (B) Relative alterations in the area under CDF curve. (C) Tracking plot showing the sample classification when k = 2–9. CDF, cumulative distribution function. [file Image_2.tif]
